# Antimicrobial potential of 1*H*-benzo[*d*]imidazole scaffold: a review

**DOI:** 10.1186/s13065-019-0521-y

**Published:** 2019-02-04

**Authors:** Sumit Tahlan, Sanjiv Kumar, Balasubramanian Narasimhan

**Affiliations:** Faculty of Pharmaceutical Sciences, Maharshi Dayanand University, Rohtak, 124001 India

**Keywords:** Benzimidazole derivatives, Antimicrobial activity, Antifungal activity

## Abstract

**Background:**

Benzimidazole is a heterocyclic moiety whose derivatives are present in many of the bioactive compounds and posses diverse biological and clinical applications. Benzimidazole agents are the vital pharmacophore and privileged sub-structures in chemistry of medicine. They have received much interest in drug discovery because benzimidazoles exhibited enormous significance. So attempts have been made to create repository of molecules and evaluate them for prospective inherent activity. They are extremely effective both with respect to their inhibitory activity and favorable selectivity ratio.

**Conclusion:**

Benzimidazole is most promising category of bioactive heterocyclic compound that exhibit a wide variety of biological activities in medicinal field. The present review only focus on antimicrobial activity of reported benzimidazole derivatives may serve as valuable source of information for researchers who wish to synthesize new molecules of benzimidazole nucleus which have immense potential to be investigated for newer therapeutic possibilities.
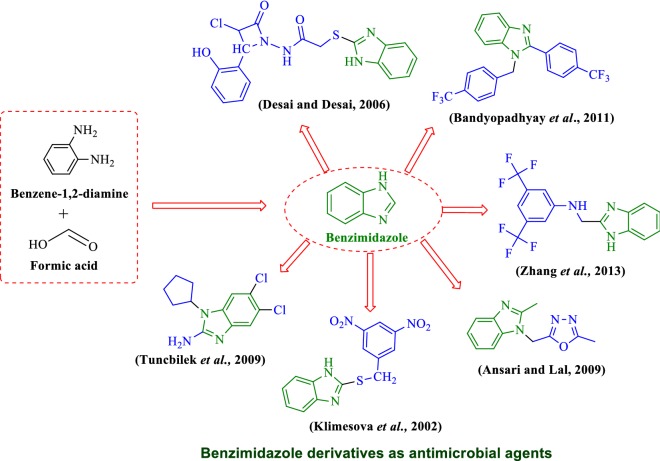

## Background

**Fig. 1:**
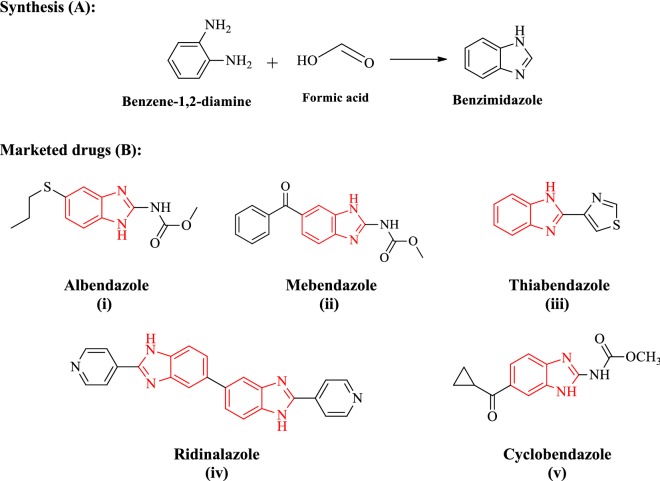
Synthesis of benzimidazole **(A)** and marketed drugs **(B)**

Benzimidazole is a dicyclic organic scaffold having imidazole (containing two nitrogen atoms at adjoining site) attached with benzene ring. Benzimidazole considered as potential bioactive heterocyclic aromatic compounds with a variety of biological activities like anti-inflammatory [1], antiparasitic [2], antimalarial [3], antimycobacterial [4], antineoplastic [5], antiviral [6], antihypertensive [7] and anticonvulsant [8] activities. Benzimidazole (synthesis (A); Fig. 1) and its derivatives are the most resourceful classes of molecules against microorganisms [9]. The increase in antimicrobial resistance to existing drugs necessitated the search for new molecules for the treatment of bacterial infections [10, 11]. Currently, a number of benzimidazole containing drugs are available in market namely: albendazole (i), mebendazole (ii), thiabendazole (iii) ridinalazon (iv) and cyclobendazole (v) (marketed drugs (B); Fig. 1).

## Biological profile

### Antimicrobial activity

**Fig. 2:**
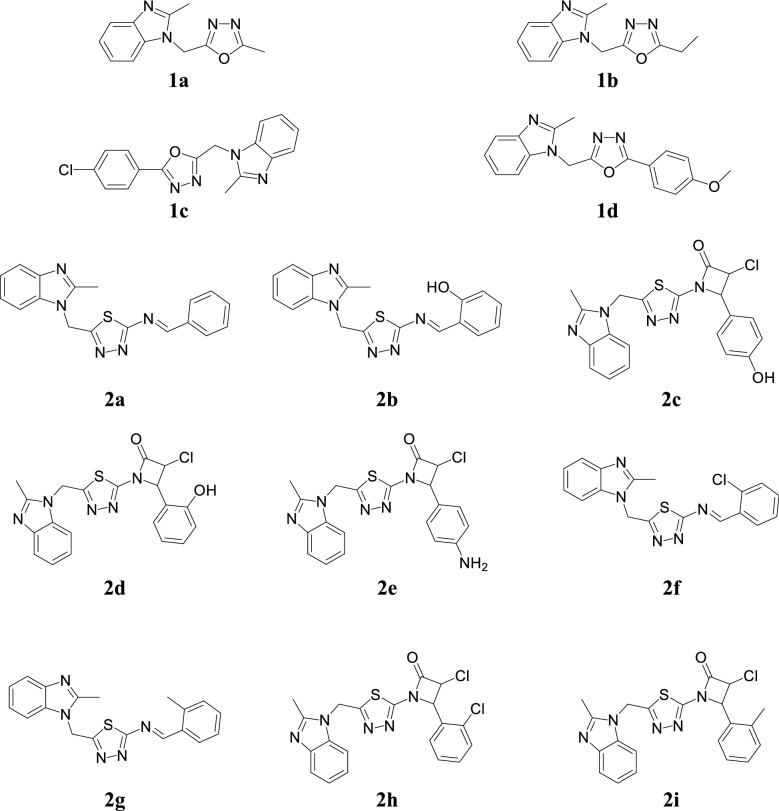
Molecular structures of compounds **(1a**–**1d, 2a**–**2i)**

Ansari et al. synthesized 2-substituted-1*H*-benzimidazole derivatives by nucleophilic substitution reaction and evaluated their antimicrobial activity against selected microbial species. The compounds **1a**, **1b**, **1c** and **1d** showed good antibacterial activity as well as compound **1c** showed good antifungal activity (Table 1, Fig. 2). SAR study inferred that at 2-position of oxadiazole ring increased side chain carbon atom number causes an enhanced the antimicrobial activity toward *C. albicans*, *S. aureus* and *B. subtilis* and also the *para*-substituted phenyl nucleus supported the activity [9].

**Table 1:** Antimicrobial activity of compounds (1a–1d)

Compounds	Antibacterial activity Microbial strains (MIC = µg/mL)						Antifungal activity (ZI mm)		
	*S. aureus*	*B. subtilis*	*S. mutans*	*E. coli*	*P. aeruginosa*	*S. typhi*	*C. albicans*	*A. niger*	*A. flavus*
**1a**	4	4	8	64	64	16	–	–	–
**1b**	4	8	4	32	> 128	32	–	–	–
**1c**	2	4	4	> 128	NE	NE	22–28	10–15	22–28
**1d**	2	8	4	16	64	16	–	–	–
**Ciprofloxacin**	≤ 1	≤ 1	NE	≤ 1	NE	NE	–	–	–
**Ampicillin**	2	2	2	4	> 128	> 128	–	–	–
**Amphotericin B**	–	–	–	–	–	–	22–28	22–28	22–28

NE: not exercised

Ansari et al. reported a series of 2-mercaptobenzimidazole derivatives and screened for its in vitro antimicrobial activity (using cup-plate agar diffusion method) against selected microbial species i.e. *E. coli*, *B. subtilis*, *A. flavus*, *C. albicans* and *A. niger.* Structure activity relationship studies revealed that compounds having *o*-Cl (**2f** and **2h**), *o*-CH_3_ (**2g** and **2i**), –OH (**2b**, **2c** and **2d**) and *p*-NH_2_ (**2e**) groups in phenyl ring as well as compound **2a** without substitution displayed significant antibacterial potential which is comparable to the reference drugs (Table 2, Fig. 2) [12].

**Table 2:** Antimicrobial activity of compounds (2a–2i)

Comp.	Microbial species	(MIC = µg/mL)					ZI mm (30 µg/mL)		
		1	10	100	200	500	*C. albicans*	*A. niger*	*A. flavus*
**2a**	*B. subtilis*	++	+	−	−	−	16–21	22–28	22–28
**2b**		++	+	−	−	−	−	−	−
**2c**		+	+	PG	−	−	16–21	16–21	16–21
**2d**		+	PG	−	−	−	−	−	−
**2e**		+	PG	−	−	−	−	−	−
**2f**	*E. coli*	++	+	−	−	−	−	−	−
**2** **g**		++	+	−	−	−	−	−	−
**2** **h**		++	+	−	−	−	−	−	−
**2i**		++	+	−	−	−	−	−	−
**Ampicillin**		+	−	−	−	−	−	−	−
**Amphotericin B**		−	−	−	−	−	22–28	22–28	22–28

Total inhibition (no growth of microorganism): (−); insufficient growth compared to control: (PG); average growth compared to control: (+); no inhibition: (++)

**Fig. 3:**
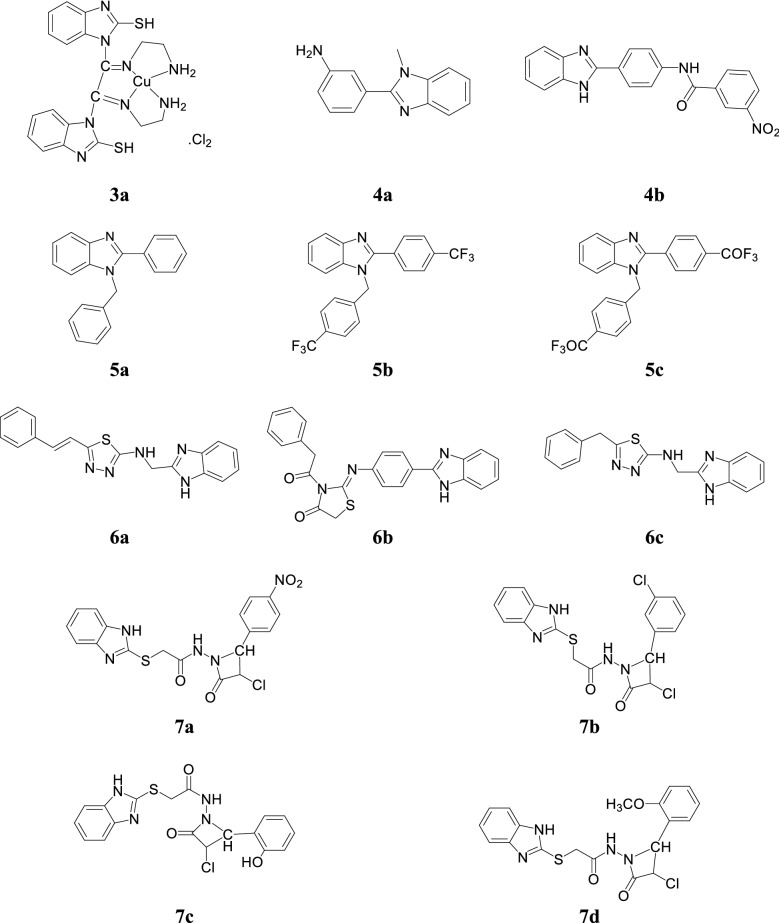
Molecular structures of compounds **(3a**, **4a**–**4b**, **5a**–**5c**, **6a**–**6c**, **7a**–**7d)**

Arjmand et al. synthesized novel Cu(II) complex benzimidazole derivative via condensation of 2-mercaptobenzimidazole with diethyloxalate and screened for their antimicrobial activity against bacterial (*E. coli, S. aureus*) and fungal (*A. niger*) species. Compound **3a** exhibited highest activity against the bacterial as well inhibited the growth of fungal species (Table 3, Fig. 3) [13].

**Table 3:** Antimicrobial activity of Cu(II) complex 3a

Compound	ZI mm (30 µg/mL)				
	Growth inhibition concentration of compound [complex] × 10^−5^ M		*S. aureus*	*E. coli*	*A. niger*
	Bacteria (*S. aureus* and *E. coli*)	Fungus (*A. niger*)			
**3a** [C_20_H_22_N_8_S_2_Cu]Cl_2_	1.7	1.7	19	17	19
	13	3.4	23	19	23
	20	5.1	25	22	25
	26	6.8	28	26	27

A novel series of benzimidazole derivatives was reported by Ayhan-Kilcigil et al. and evaluated for its antimicrobial potential against selected strains by the tube dilution technique. Compound, **4a** showed significant antimicrobial potential against *B. subtilis* and *P. aeruginosa* with MIC values of 12.5 and 25 µg/mL, respectively which is comparable to ampicillin (MIC = 6.25 and 25 µg/mL) as well **4a** and **4b** (Fig. 3) showed good antifungal activity with MIC values of 6.25 and 12.5 µg/mL (*C. albicans*) as comparable with fluconazole (MIC = 6.25 µg/mL) and miconazole (MIC = 3.125 µg/mL) [14].

Bandyopadhyay et al. synthesized new class of 1,2-disubstituted benzimidazole derivatives using Al_2_O_3_–Fe_2_O_3_ nanocrystals as heterogeneous catalyst under mild reaction conditions and evaluated for its antibacterial activity (Kirby–Bauer disc diffusion method) against *B. cereus*, *V. cholerae*, *S. dysenteriae, S. aureus* and *E. coli*. Compounds, **5a, 5b** and **5c** (Fig. 3) showed good activity as compared to standard ciprofloxacin. Additionally, compounds **5a** and **5c** showed absolute bactericidal activity against tested strains within 24 h, whereas ciprofloxacin kill those bacteria in 48 h (Table 4) [15].

**Table 4:** Antibacterial activity of compounds (5a–5c)

Comp.	Microorganisms (ZI mm)				
	*E. coli*	*V. cholerae*	*S. dysenteriae*	*S. aureus*	*B. cereus*
**5a**	19	33	23	10	22
**5b**	22	13	19	22	–
**5c**	–	23	11	10	–
**Ciprofloxacin**	32	24	14	15	14

Barot et al. developed some novel benzimidazole derivatives and evaluated for their antimicrobial potential towards *P. aeruginosa*, *E. coli*, *B. cereus*, *K. pneumonia*, *S. aureus*, *E. faecalis*, *C. albicans*, *A. niger* and *F. oxyspora* and compared to standard drugs ofloxacin metronidazole and fluconazole. From this series, compounds **6a** and **6b** revealed good antibacterial activity where as compound **6c** showed significant antifungal activity (Table 5, Fig. 3) [10].

**Table 5:** Antimicrobial activity of compounds (6a–6c)

Comp.	Microorganisms (MIC in µg/mL)								
	*B. cereus*	*E. faecalis*	*S. aureus*	*E. coli*	*P. aeruginosa*	*K. pneumonia*	*C. albicans*	*A. niger*	*F. oxyspora*
**6a**	5	7	7	10	10	9	–	–	–
**6b**	5	7	8	8	8	11	–	–	–
**6c**	–	–	–	–	–	–	8	7	8
**Ofloxacin**	2	2	3	4	4	5	–	–	–
**Metronidazole**	3	3	3	3	4	4	–	–	–
**Fluconazole**	–	–	–	–	–	–	2	3	3

Desai et al. reported a series of 2-mercaptobenzimidazole and *β*-lactum segment derivatives containing –CONH– and evaluated for its in vitro antibacterial (Kirby–Bauer disc diffusion technique) and antifungal potentials against tested microorganisms using streptomycin and flucanozole as standards. Among the synthesized compounds, **7a** displayed tremendous inhibitory activity against *B. subtilis*, **7b** showed excellent activity against *E. coli* and *S. aureus*, **7c** showed considerable activity against *A. niger* and **7d** showed significant activity against *C. krusei* (Table 6, Fig. 3) [16].

**Table 6:** Antimicrobial activity results of compounds (7a–7d)

Compounds	Microorganisms					
	Bacteria (ZI mm)			Fungi (MIC = µg/mL)		
	*B. subtilis*	*S. aureus*	*E. coli*	*C. albicans*	*C. krusei*	*A. niger*
**7a**	20–25	15–20	15–20	–	–	–
**7b**	15–20	20–25	20–25	–	–	–
**7c**	–	–	–	150	100	150
**7d**	–	–	–	150	150	100
**Streptomycin**	25–30	25–30	25–30	–	–	–
**Fluconazole**	–	–	–	50	50	50

**Fig. 4:**
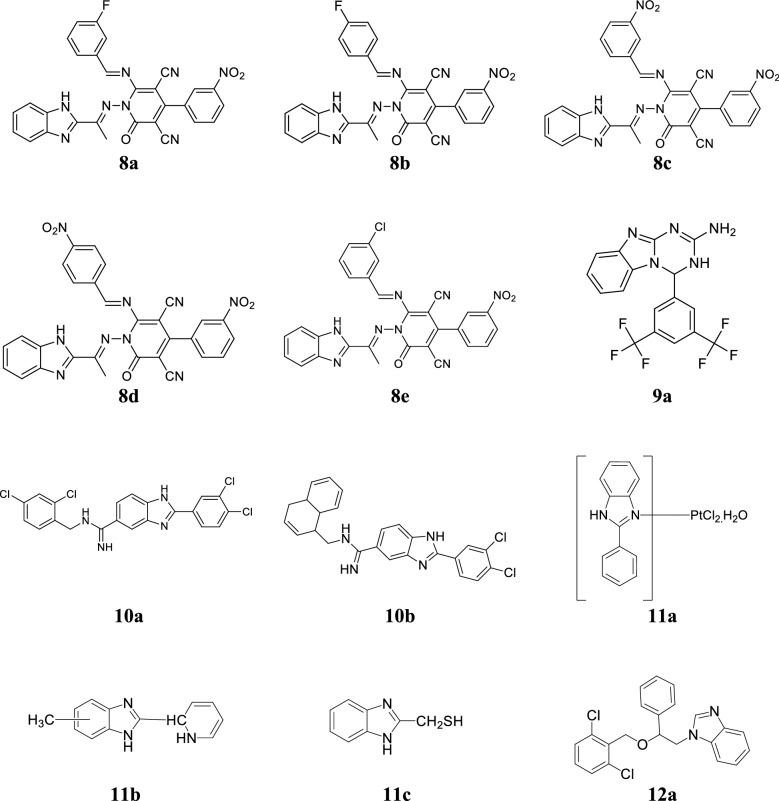
Molecular structures of compounds **(8a**–**8e**, **9a**, **10a**–**10b**, **11a**–**11c**, **12a)**

Desai et al. reported new benzimidazoles bearing 2-pyridone and evaluated for their antimicrobial activity against *S. pyogenes*, *E. coli*, *S. aureus*, *P. aeruginosa*, *C. albicans*, *A. clavatus* and *A. niger* by conventional broth dilution technique. Among the synthesized compounds, **8a**, **8b**, **8c** and **8d** (Table 7, Fig. 4) having electron withdrawing group (nitro) at the *m*-position enhanced the antibacterial activity and compared to chloramphenicol while compound **8e** displayed most effective antifungal activity and comparable to standard ketoconazole [11].

**Table 7:** Antimicrobial activity results of compounds (8a–8e)

Comp.	Microorganisms (MIC = µg/mL)						
	*S. aureus*	*S. pyrogens*	*E. coli*	*P. aeruginosa*	*C. albicans*	*A. niger*	*A. clavatus*
**8a**	12.5 ± 1.05	12.5 ± 1.21	25 ± 1.35	25 ± 2.80	500 ± 1.57	100 ± 1.24	250 ± 2.78
**8b**	50 ± 1.54	50 ± 1.31	100 ± 2.65	100 ± 1.61	500 ± 2.15	250 ± 2.21	250 ± 1.24
**8c**	12.5 ± 1.48	25 ± 2.15	25 ± 1.35	25 ± 1.15	100 ± 1.64	500 ± 1.85	250 ± 1.32
**8d**	25 ± 1.21	50 ± 1.81	25 ± 1.54	50 ± 1.51	250 ± 1.32	> 1000	500 ± 2.32
**8e**	62.5 ± 1.35	100 ± 1.65	125 ± 1.42	125 ± 1.71	25 ± 1.41	50 ± 1.14	62.5 ± 1.35
**Chloram-phenicol**	50 ± 1.24	50 ± 2.04	50 ± 1.00	50 ± 2.06	–	–	–
**Ketoconazole**	–	–	–	–	50 ± 0.50	50 ± 1.20	50 ± 1.10

Dolzhenko et al. prepared novel 3,4-dihydro [1, 3, 5] triazino[1,2-*a*]benzimidazole compounds and screened for their in vitro antibacterial activity by twofold serial dilution technique. Compound **9a** exhibited good antibacterial potential as compared to standard drug tetracyclin (Table 8, Fig. 4**)** [17].

**Table 8:** Antibacterial activity of the fluorinated compound 9a

Compound	Microbial strains (MIC = µg/mL)				
	*S. aureus*	*B. subtilis*	*B. megaterium*	*K. aerogenes*	*E. coli*
**9a**	25	25	>25	> 25	> 25
**Tetracycline**	0.63	0.63	0.63	1.25	1.25

Goker et al. developed novel substituted benzimidazole carboxamidine molecules and assessed for their antibacterial activity by tube dilution method against selected microbes. Compounds **10a** and **10b** displayed significant antibacterial activity (Table 9, Fig. 4) as comparable to standard drugs (ampicillin and sultamicillin) [18].

**Table 9:** In vitro antibacterial activity of compounds (10a–10b)

Compounds	Microorganisms MIC (µg/mL)			
	*S. aureus*	MRSA	MRSA (isolate from blood)	MRSA (isolate from wound)
**10a**	0.78	0.78	0.39	1.56
**10b**	0.39	0.78	0.39	0.78
**Ampicillin**	0.78	50	50	50
**Sultamicillin**	0.39	25	25	25

Gumus et al. synthesized platinum(II) complexes with substituted benzimidazole ligands and evaluated for their antimicrobial potential against *S. aureus*, *P. aeruginosa, S. faecalis*, *E. coli* and *C. albicans* using the macro dilution broth method. Complex **11a** (MIC = 100 µg/mL) exhibited good antibacterial activity against *S*. *faecalis*, **11b** (Mpyrb- methyl *α*-pyridyl benzimidazole, MIC = 50 µg/mL) against *C*. *albicans* and **11c** (Merb- mercaptobenzimidazole, MIC = 50 and 100 µg/mL) (Fig. 4) found active against *S*. *faecalis* and *S*. *aureus* [19].

Guven et al. reported a new class of benzimidazole and phenyl-substituted benzyl ethers and evaluated for its antimicrobial potential against selected microbial species. Among the synthesized derivatives, compound **12a** (Table 10, Fig. 4) exhibited good antibacterial activity and comparable to the standard drug [20].

**Table 10:** In vitro antimicrobial activity of compounds (12a)

Compounds	Microbial strains MIC (µg/mL)			
	*S. aureus*	MRSA	*C. albicans*	*C. krusei*
**12a**	3.12	6.25	12.5	12.5
**Ampicillin**	0.78	25	–	–
**Fluconazole**	–	–	0.78	25
**Miconazole**	–	–	0.19	0.78

**Fig. 5:**
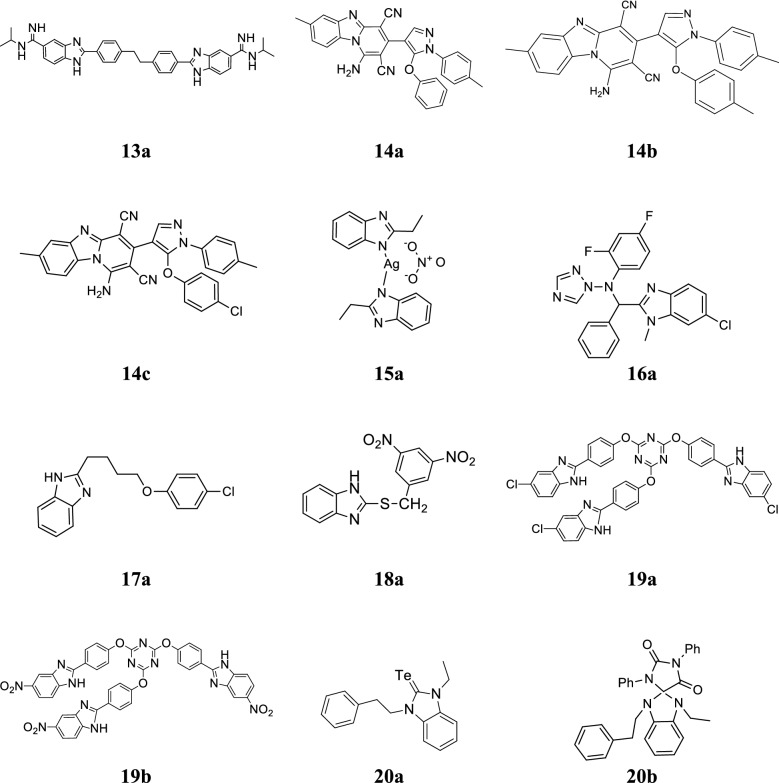
Molecular structures of compounds **(13a**, **14a**–**14c**, **15a**, **16a**, **17a**, **18a**, **19a-19b**, **20a–20b)**

Hu et al. designed new bis-benzimidazole diamidine compounds and evaluated for their antibacterial activity against tested species and compared to standard drugs (penicillin G, vancomycin and ciprofloxacin). Compound **13a** exhibited the potent antibacterial activity than vancomycin (Table 11, Fig. 5) [21].

**Table 11:** Antibacterial results of compound 13a

Compound	Strains	MIC (µg/mL)	Penicillin-G	Ciprofloxacin	Vancomycin
**13a**	*S. aureus*	0.25–0.5	1	0.5	0.5
	*S. aureus* ^a^	0.5	> 32	8	1
	*S. aureus* ^b^	0.25–0.5	> 32	≤ 0.12	1
	*S. epidermidis*	< 0.06	32	≤ 0.12	1
	*S. epidermidis* ^c^	0.125	32	≤ 0.12	1
	*S. pneumoniae*	< 0.06	< 0.06	0.5	1
	*E. faecalis* ^d^	0.25–0.5	4	0.5	> 64
	*E. faecium* ^d^	0.12	> 32	> 64	> 64
	*B. subtils*	0.12	< 0.06	≤ 0.12	0.12–0.5
	*B. cereus*	0.12	4– > 32	≤ 0.12	1– ≤ 0.12
	*B. fragile*	0.5–1	4–8	0.5	4–8
	*C. perfringens*	0.25–0.5	≤ 0.06–0.12	0.25	0.12–0.25

^a^MDRSA

^b^MRSA

^c^MRSE

^d^VRE

Jardosh et al. developed a novel series of pyrido[1,2-*a*]benzimidazole derivatives and assessed for its in vitro antimicrobial activity against *S. typhi*, *S. pneumoniae*, *E. coli*, *C. tetani*, *V. cholera*, *B. subtilis*, *C. albicans* and *A. fumigatus* using broth micro dilution technique. Among the synthesized derivatives, compounds **14a**–**14c** (Fig. 5) displayed the good antimicrobial activity and compared to standard drugs (Table 12, Fig. 5**)** [22].

**Table 12:** In vitro antimicrobial activity of benzimidazole compounds (14a–14c)

Compounds	Microorganisms (MIC = µg/mL)							
	*B. subtilis*	*C. tetani*	*S. pneumoniae*	*E. coli*	*S. typhi*	*V. cholera*	*A. fumigatus*	*C. albicans*
**14a**	100	200	100	200	250	250	> 1000	250
**14b**	500	200	200	250	250	50	200	> 1000
**14c**	250	250	250	62.5	200	100	> 1000	250
**Ciprofloxacin**	50	100	50	25	25	25	–	–
**Chloramphenicol**	50	50	50	50	50	50	–	–
**Norfloxacin**	100	50	10	10	10	10	–	–
**Ampicillin**	250	250	100	100	100	100	–	–
**Griseofulvin**	–	–	–	–	–	–	100	500

Kalinowska-Lis et al. synthesized silver (I) complexes of benzimidazole and screened for their antimicrobial activity against *S. epidermidis*, *S. aureus* and *C. albicans*. In this series, compound **15a** (Fig. 5) exhibited good antifungal but moderate antibacterial activity as compared to standard drugs AgNO_3_ and silver sulfadiazine (AgSD) (Table 13) [23].

**Table 13:** Antimicrobial activity results of compound 15a

Compound 15a	Microorganisms											
	*S. aureus*				*S. epidermis*				*C. albicans*			
	MIC		MBC		MIC		MBC		MIC		MBC	
	mg/L	µM/L	mg/L	µM/L	mg/L	µM/L	mg/L	µM/L	mg/L	µM/L	mg/L	µM/L
**[Ag(2-CH**_**2**_ **OHbim)**_**2**_ **]NO**_**3**_	80	171	90	193	80	171	90	193	10	20	20	43
**AgNO**_**3**_	15	88	25	147	15	88	20	118	10	59	30	117
**Silver sulfadiazine (AgSD)**	60	168	90	252	40	112	80	224	20	56	20	56

Kankate et al. developed novel benzimidazole analogues and screened for their in vitro (tube dilution technique) and in vivo antifungal activity (kidney burden test) against *C. albicans*. Compound **16a** (Fig. 5) exhibited superior in vitro antifungal activity with MIC value of 0.0075 µmol/mL as comparable to fluconazole while in vivo activity was significantly less (P < 0.001) [24].

Khalafi-Nezhad et al. synthesized some chloroaryloxyalkyl benzimidazole derivatives and screened for their in vitro antimicrobial activity against *S. typhi* and *S. aureus* using disk diffusion method. Compound **17a** showed good antibacterial activity against the tested microbial species (Table 14, Fig. 5) [25].

**Table 14:** Antibacterial screening results of compound 17a

Compound	Microorganisms (MIC = µg/mL)	
	*S. aureus*	*S. typhi*
**17a**	22	24
**Chloramphenicol**	16	20
**Hexachlorophene**	10	1

Klimesova et al. developed a chain of 2-alkylsulphanylbenzimidazoles and evaluated for its in vitro antimycobacterial and antifungal activities against selected strains using isoniazide and ketoconazole as standards. Among the synthesized compounds, **18a** exhibited significant antimycobacterial and antifungal activities (Table 15, Fig. 5) [26].

**Table 15:** Antimycobacterial screening results of compound 18a (MIC = µmol/L)

Compound	Bacterial strains										Fungal strains		
	*M. tuberculosis* MY 331/88		*M. kansasii* My 235/80			*M. kansasii* My 6509/96			*M. avium* (*M.* My 330/88)		*T. mentagrophytes* 445	*A. corymbifera* 272	*A. fumigates* 231
	14 days	21 days	7 days	14 days	21 days	7 days	14 days	21 days	14 days	21 days	72 h	24 h	24 h
**18a**	4	4	4	8	8	4	8	8	8	8	62	–	–
**Isoniazide**	0.5	1	> 250	> 250	> 250	2	4	4	> 250	> 250	–	–	–
**Ketoconazole**	–	–	–	–	–	–	–	–	–	–	0.98	31.25	7.81

Koc et al. synthesized few tripodal-benzimidazole derivatives and evaluated for their antibacterial activity against *S. aureus*, *B. subtilis* and *E. coli* by standard disk diffusion technique using gentamycin as reference. Among the synthesized compounds, **19a** and **19b** exhibited good antibacterial activity toward *E. coli*, *S. aureus* and *B. subtilis* (Table 16, Fig. 5) [27].

**Table 16:** Antimicrobial activity of compounds (19a–19b)

Compounds	Microorganisms (ZI/mm^2^)		
	*E. coli*	*B. subtilis*	*S. aureus*
**19a**	7	9	9
**19b**	7	9	10
**Gentamycin**	16	16	18

Kucukbay et al. synthesized new electron-rich olefins benzimidazole compounds and evaluation for their in vitro antimicrobial activity against the selected microbial species and compared to standard drug. Among the prepared compounds, **20a** and **20b** were found to be most effective against *C. albicans* and *C. tropicalis* (Table 17, Fig. 5) [28].

**Table 17:** Antimicrobial results of compounds (20a–20b)

Compound	Microorganisms (MIC = µg/mL)					
	Bacteria				Fungi	
	*E. Faecalis*	*S. aureus*	*E. coli*	*P. aeruginosa*	*C. albicans*	*C. tropicalis*
**20a**	200	200	50	50	–	–
**Ampicillin**	0.78	0.39	3.12	> 75	–	–
**20b**	–	–	–	–	50	50
**Fluconazole**	–	–	–	–	1.25	1.25

**Fig. 6:**
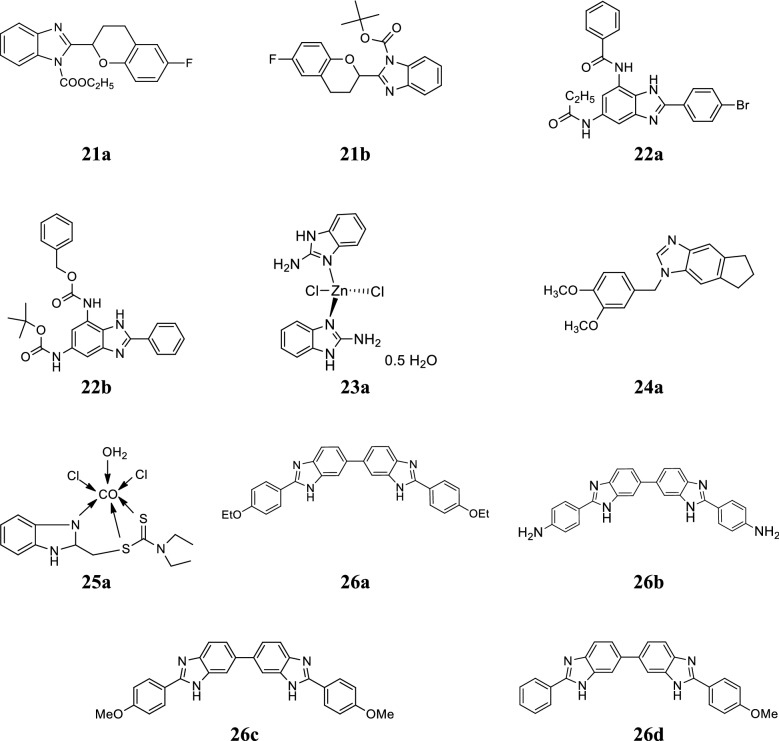
Molecular structures of compounds **(21a**–**21b**, **22a**–**22b**, **23a**, **24a**, **25a**, **26a**–**26d)**

Kumar et al. developed a new series of substituted benzimidazole scaffolds and screened for its in vitro antibacterial potential against *S. aureus* and *S. typhimurium* and compared to cephalexin as standard. Compounds, **21a** and **21b** exhibited good antibacterial activity against *S. typhimurium* whereas showed pitiable activity against *S. aureus* (Table 18, Fig. 6) [29].

**Table 18:** Antibacterial activity of compounds (21a–21b)

Compounds	Concentration (µg/mL) (*S. typhimurium*)						
	0.1	1	10	100	200	500	App. MIC
**21a**	+	+	PG	PG	−	−	200
**21b**	+	+	+	PG	−	−	200
**Cephalexin**	++	++	+	PG	−	−	200

Full inhibition, no growth of organism: −; meager growth compared to controls: PG; average growth compared to controls: +; confluent growth, inhibition: ++

Kumar et al. reported a series of trisubstituted benzimidazole molecules and screened for its antimicrobial potential against *F. tularensis* LVS strain using Microplate Alamar Blue assay. Compounds, **22a** and **22b** (Fig. 6) exhibited promising antimicrobial activity with MIC values of 0.35 and 0.48 µg/mL [30].

Lopez-Sandoval et al. reported a series of cobalt (II) and zinc (II) coordination complexes with benzimidazole and evaluated for its antimicrobial potential by disk diffusion method and antibiotics microbial assays (U.S.P 23) against *P. aeruginosa*, *E. coli*, *S. typhi*, *M. luteus*, *S. aureus* and *P. vulgaris*. Among the synthesized complexes, complex **23a** exhibited good activity toward *M. luteus* and *E. coli* (Table 19, Fig. 6) [31].

**Table 19:** Antibacterial activity of compound 23a

Compound 23a	Microorganisms			
	*M. luteus*		*E. coli*	
	ZI (mm)	MIC (µg/mL)	ZI (mm)	MIC (µg/mL)
**[Zn(2aminobenzimidazole)**_**2**_ **Cl**_**2**_ **]·0.5H**_**2**_ **O**	10	1.6	11.1	3.9
**Amoxicillin**	10.4	0.125	–	–
**Chloramphenicol**	–	–	11.3	1.6

Mehboob et al. reported a class of second generation benzimidazole derivatives and screened for its antibacterial activity against *S. aureus*, MRSA, *F. tularensis* and *E. coli*. Among the synthesized compounds, **24a** exhibited good antibacterial activity against selected bacterial strains (Table 20, Fig. 6) [32].

**Table 20:** Compound 24a MIC/MBC (µg/mL) values of compound 24a

Compound	Microorganisms				
	*F. tularensis*	*S. aureus*	MRSA	*E. coli*	*E. coli* TolC-
**24a**	5.5/12.5	> 12.5	> 12.5	> 12.5	12.5

MBCs were not determined for compounds with MICs ≥ 12.5 µg/mL. *E. coli* TolC- is the *E. coli* TolC efflux pump knockout mutant

Mohamed et al. reported a class of seven transition metal complexes of benzimidazole and assessed for its antifungal activity against *F. solani*, *R. solani* and *S. rolfesii*. Among the synthesized metal complexes, cobalt complex **25a** (Fig. 6) displayed the highest fungicidal activity with lowest EC_50_ values of 353.55, 205.45 and 196.84 ppm for the *F. solani*, *R. solani* and *S. rolfesii*, respectively [33].

Moreira et al. reported a series of bis-benzimidazole conjugates and screened for its antibacterial activity against selected microbes. Among the synthesized derivatives, compounds **26a**, **26b** and **26c** possessed excellent activity against Gram-positive bacteria with MIC_90_ values between 0.06 and 1 mg/L. Compounds **26c** and **26d** exhibited significant activity against *M. tuberculosis* H37Rv with MIC value of 2 mg/L and 1 mg/L, respectively (Fig. 6) [34].

**Fig. 7:**
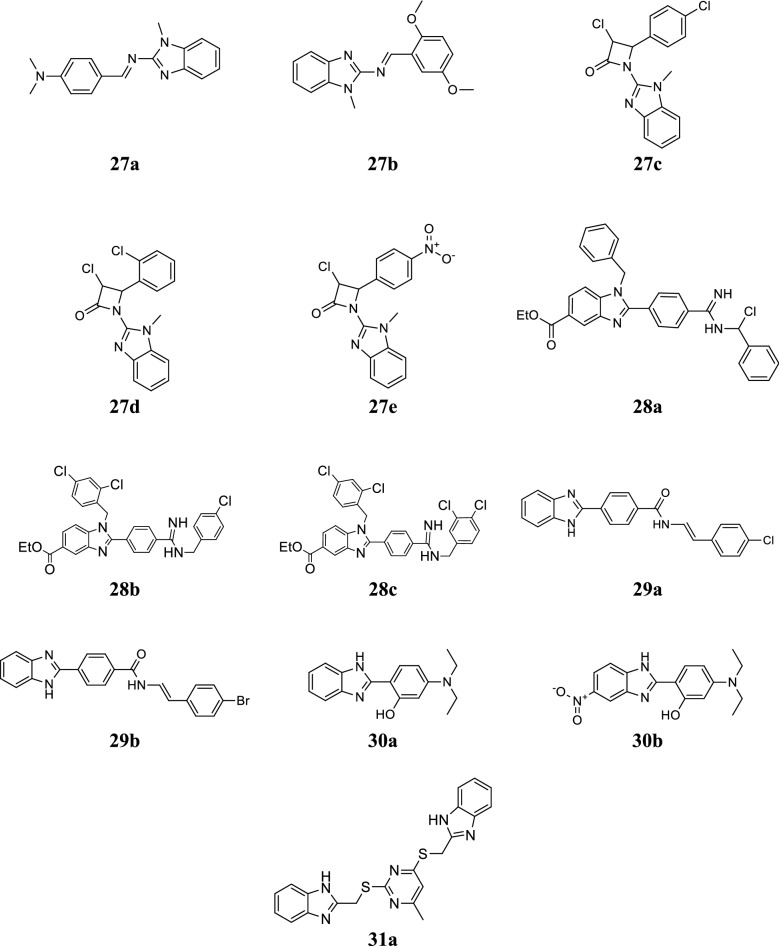
Molecular structures of compounds **(27a**–**27e**, **28a**–**28c**, **29a**–**29b**, **30a**–**30b**, **31a)**

Noolvi et al. developed a class of 1*H*-benzimidazole azetidine-2-one scaffolds and assessed for its antibacterial activity against selected bacteria (*S. aureus*, *B. pumillus*, *E. coli* and *P. aeruginosa*). The MIC and ZI of the synthesized compounds was determined by agar diffusion technique. Compounds **27a**–**27e** showed significant antibacterial activity as comparable to ampicillin (Table 21, Fig. 7) [35].

**Table 21:** In vitro antimicrobial activity of compounds (27a–27e)

Compounds	Microorganisms (ZI mm)				Microorganisms (MIC = µg/mL)			
	*S. aureus*	*B. pumillus*	*E. coli*	*P. aeruginosa*	*S. aureus*	*B. pumillus*	*E. coli*	*P. aeruginosa*
**27a**	11.3	10.2	10.8	10.6	–	–	–	–
**27b**	10.9	10.5	11.2	11.0	–	–	–	–
**27c**	13.2	11.2	13.6	10.9	–	–	–	–
**27d**	13.2	11.5	12.8	11.3	25	25	50	75
**27e**	–	–	–	–	25	25	50	50
**Ampicillin**	14.8	12.8	15.2	13.4	6.5	12.5	25	25

Ozden et al. synthesized a chain of benzimidazole-5-carboxylic acid alkyl esters and evaluated for its antimicrobial activity against methicillin resistant *E. coli*, MRSA, *S. aureus*, *S. faecalis*, MRSE and *C. albicans*. Compounds **28a**, **28b** and **28c** exhibited promising antimicrobial activity as compared to reference drugs (Table 22, Fig. 7) [36].

**Table 22:** Antibacterial and antifungal activities of compounds (28a–28c)

Compounds	Minimum inhibitory concentration (µg/mL)					
	*S. aureus*	MRSA	*S. faecalis*	MRSE	*E. coli*	*C. albicans*
**28a**	0.78	0.78	6.25	1.56	> 50	12.5
**28b**	1.56	0.78	3.12	0.78	> 50	12.5
**28c**	1.56	0.39	3.12	1.56	> 50	6.25
**Ampicillin**	0.39	50	0.78	–	–	–
**Sultamicillin**	0.78	25	1.56	3.12	–	–
**Gentamisin**	–	–	–	–	0.78	–
**Fluconazole**	–	–	–	–	–	1.56

Ozkay et al. developed a series of benzimidazole compounds with hydrazone moiety and assessed for its in vitro antimicrobial potential against bacterial (*E. faecalis*, *B. subtilis, L. cytogenes, S. aureus*, *P. aeruginosa*, *K. pneumoniae*, *E. coli* ATCC 35218, *E. coli* ATCC 25922, *S. typhimurium, P. vulgaris*) and fungal (*C. albicans*, *C. tropicalis*, *C. globrata*) species by twofold serial dilutions technique taking chloramphenicol and ketocanozole as reference drugs. In this series, compounds, **29a** and **29b** showed promising antibacterial and antifungal activities as compared to standard drugs (Tables 23 and 24, Fig. 7) [37].

**Table 23:** MIC values (µg/mL) of compounds (29a–29b) against Gram-negative bacteria

Compound	Microorganisms					
	*E. coli* ATCC 35218	*E. coli* ATCC 25922	*P. vulgaris*	*S. typhimurium*	*K. pneumoniae*	*P. aeruginosa*
**29a**	25	100	25	6.25	12.5	25
**29b**	25	50	25	12.5	12.5	25
**Chloramphenicol**	12.5	12.5	50	12.5	12.5	50

**Table 24:** MIC values (µg/mL) of compounds (29a–29b) against Gram-positive bacteria and fungal strains

Compounds	Microorganisms						
	*L. monocytogenes*	*S. aureus*	*E. faecalis*	*B. subtilis*	*C. albicans*	*C. globrata*	*C. tropicalis*
**29a**	100	12.5	12.5	25	50	50	50
**29b**	200	25	12.5	25	100	100	50
**Chloramphenicol**	50	12.5	12.5	12.5	–	–	–
**Ketoconazole**	–	–	–	–	50	25	50

Padalkar et al. synthesized a new class of 2-(1*H*-benzimidazol-2-yl)-5-(diethylamino) phenol derivatives and screened for its antimicrobial potential against *S. aureus*, *E. coli*, *A. niger* and *C. albicans* using serial dilution method. Among them, compounds, **30a** (2-(1*H*-benzo[*d*]imidazol-2-yl)-5-(diethylamino)phenol) and **30b** (5-(diethylamino)-2-(5-nitro-1*H*-benzo[*d*]imidazol-2-yl)phenol) displayed significant activity against tested bacterial species and their activity results are similar to the reference drug (Table 25, Fig. 7) [38].

**Table 25:** Antimicrobial activity of compounds (30a–30b)

Compounds	Microorganisms [MIC (µg/mL)]			
	*E. coli*	*S. aureus*	*C. albicans*	*A. niger*
**30a**	60	60	130	130
**30b**	60	60	130	250
**Streptomycin**	60	60	–	–
**Fluconazole**	–	–	60	60

Seenaiah et al. reported a series of benzimidazole derivatives and screened for its antimicrobial activity against selected bacterial and fungal species by agar well diffusion (ZI) and broth dilution methods (MIC). In this series, compound **31a** displayed promising activity against tested microorganisms as comparable to standard drugs (Tables 26, 27, 28 and Fig. 7) [39].

**Table 26:** Antimicrobial activity of compound 31a

Compound	ZI (mm)								
	Gram + ve			Gram − ve					
	*S. aureus*			*E. coli*			*P. aeruginosa*		
	25 µg/mL	50 µg/mL	100 µg/mL	25 µg/mL	50 µg/mL	100 µg/mL	25 µg/mL	50 µg/mL	100 µg/mL
**31a**	23 ± 3	26 ± 1	29 ± 2	28 ± 1	32 ± 4	35 ± 3	22 ± 1	25 ± 3	27 ± 2
**Ciprofloxacin**	22 ± 1	24 ± 3	27 ± 1	30 ± 2	35 ± 3	38 ± 2	25 ± 1	28 ± 2	30 ± 3

**Table 27:** Antifungal activity of compound 31a

Compound	Fungus (ZI mm)					
	*A. niger*			*P. chrysogenum*		
	25 µg/mL	50 µg/mL	100 µg/mL	25 µg/mL	50 µg/mL	100 µg/mL
**31a**	27 ± 1	30 ± 3	32 ± 1	33 ± 2	35 ± 1	38 ± 2
**Ketoconazole**	31 ± 2	33 ± 3	36 ± 3	35 ± 1	36 ± 2	38 ± 3

**Table 28:** Antimicrobial activity of compound 31a

Compound	MIC (MBC/MFC) µg/mL				
	*S. aureus*	*E. coli*	*P. aeruginosa*	*A. niger*	*P. chrysogenum*
**31a**	12.5 (25)	50 (200)	12.5 (100)	12.5 (100)	12.5 (25)
**Ciprofloxacin**	12.5	12.5	12.5	–	–
**Ketoconazole**	–	–	–	6.25	12.5

**Fig. 8:**
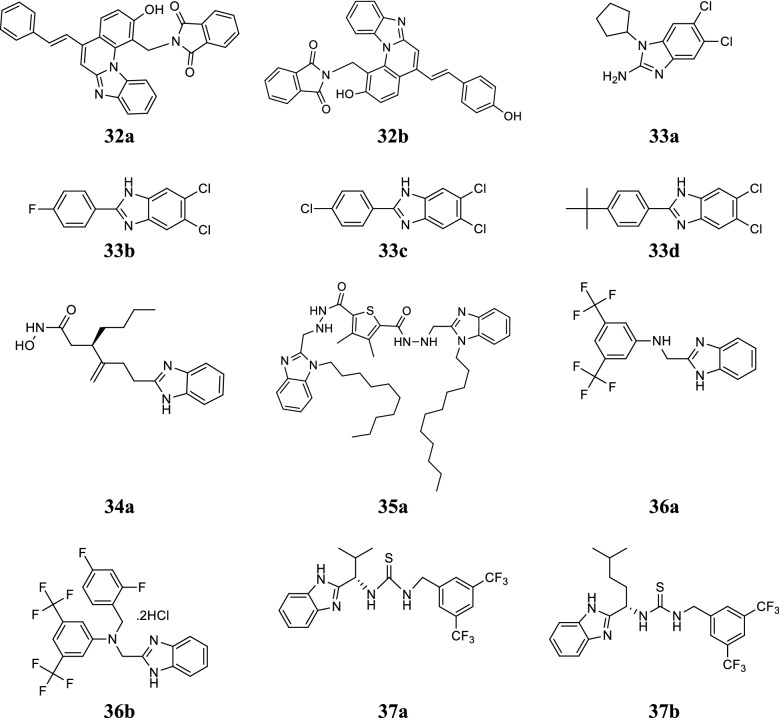
Molecular structures of compounds **(32a**–**32b**, **33a**–**33d**, **34a**, **35a**, **36a**–**36b**, **37a**–**37b)**

Tiwari et al. designed a new series of benzimidazole scaffolds and evaluated for its in vitro antifungal potential against *A. flavus* and *A. niger* by agar plate method. From the synthesized derivatives, compounds **32a** and **32b** showed excellent antimicrobial activity as comparable to reference (amphotericin B) (Table 29, Fig. 8) [40].

**Table 29:** Antifungal activity of benzimidazole derivatives (32a–32b)

Compounds	Concentration (µg/mL)	Microorganisms			
		*A. flavus*		*A. niger*	
		Colony diameter	Inhibition (%)	Colony diameter	Inhibition (%)
**32a**	10	0.8	73.3	1.0	60.3
	20	0.6	76.7	0.8	76.8
	50	0.5	88.3	0.5	84.6
**32b**	10	1.2	60.8	0.8	60.7
	20	1.1	73.4	0.7	83.2
	50	0.7	92.1	0.7	83.6
**Amphotericin B**	20	3.0	86.4	2.0	79.9

Tuncbilek et al. designed some novel benzimidazole derivatives and screened for their antimicrobial potential toward *E. coli*, *B. subtilis*, MRSA (clinical and standard isolates), *S. aureus* and *C. albicans*. Compounds **33a**–**33d** displayed the excellent antibacterial activity as comparable to reference drugs (sultamicillin, ciprofloxacin and ampicillin) (Table 30, Fig. 8) [41].

**Table 30:** Antibacterial and antifungal activities of compounds (33a–33d)

Compounds	Microorganisms (MIC = µg/mL)					
	*S. aureus*	MRSA^a^	MRSA^b^	*E. coli*	*B. subtilis*	*C. albicans*
**33a**	3.12	6.25	6.25	50	6.25	6.25
**33b**	3.12	3.12	3.12	50	6.25	6.25
**33c**	3.12	3.12	3.12	50	50	12.5
**33d**	3.12	3.12	3.12	50	6.25	12.5
**Sultamicillin**	0.39	25	25	–	0.78	–
**Ampicillin**	0.78	50	50	–	–	–
**Ciprofloxacin**	0.78	6.25	12.5	0.19	0.09	–
**Fluconazole**	–	–	–	–	–	1.56

^a^MRSA—standard

^b^MRSA—clinical isolate

Zhang et al. synthesized a chain of new actinonin derivatives of benzimidazole and evaluated for its antimicrobial potential against *S. lutea*, *K. pneumoniae* and *S. aureus* using microbroth dilution method. Compound **34a** ((*R*)-3-(4-(1*H*-benzo[*d*]imidazol-2-yl)but-1-en-2-yl)-*N*-hydroxy heptanamide) showed potent antibacterial activity against tested microorganism than the standard drug (Table 31, Fig. 8) [42].

**Table 31:** Antibacterial activity of compound 34a

Compound	Microorganisms (MIC = µg/mL)		
	*S. aureus*	*K. pneumonia*	*S. lutea*
**34a**	2	0.5	4
**Cefoperazone**	0.25	0.25	0.25

Zhang et al. reported a class of substituted benzimidazole compounds and screened for its antimicrobial potential against two fungal, four Gram-positive and five Gram-negative bacterial strains through twofold serial dilution technique. Among them, compound **35a** exhibited remarkable antimicrobial activity even better than the standards fluconazole, chloromycin and norfloxacin (Tables 32, 33 and Fig. 8) [43].

**Table 32:** Antibacterial and antifungal activities of compound 35a

Compound	Microorganisms (MIC = µg/mL)					
	Bacteria (Gram + ve)				Fungi	
	MRSA	*S. aureus*	*B. subtilis*	*M. luteus*	*C. albicans*	*C. mycoderma*
**35a**	2	2	4	16	4	2
**Chloromycin**	16	16	32	8	–	–
**Norfloxacin**	8	0.5	1	2	–	–
**Fluconazole**	–	–	–	–	1	4

**Table 33:** Antibacterial activity (MIC = µg/mL) of compound 35a

Compound	Microorganisms (Gram − ve bacteria)				
	*E. coli*	*S. dysenteriae*	*P. aeruginosa*	*B. proteus*	*E. typhosa*
**35a**	4	8	4	8	4
**Chloromycin**	32	32	32	32	32
**Norfloxacin**	16	4	16	8	4

Zhang et al. designed a novel class of benzimidazole type of fluconazole compounds and evaluated for its antimicrobial activity by two-fold serial dilution technique. Among them, compounds **36a** and **36b** exhibited the potent antimicrobial efficiency as compared to standards norfloxacin, chloromycin and fluconazole (Tables 34 and 35, Fig. 8) [44].

**Table 34:** Antibacterial activity (MIC = µg/mL) of compounds (36a–36b)

Compounds	Microorganisms (bacteria)							
	*S. aureus*	MRSA (N315)	*B. subtilis*	*M. luteus*	*B. proteus*	*E. coli*	*P. aeruginosa*	*B. typhi*
**36a**	2	16	4	8	2	2	4	2
**36b**	8	16	8	8	16	32	16	16
**Chloromycin**	8	16	32	8	32	16	16	32
**Norfloxacin**	1	2	1	4	1	1	1	1

**Table 35:** Antifungal activity (MIC = µg/mL) of compound 36a

Compound	Microorganisms (fungi)				
	*C. albicans*	*C. mycoderma*	*C. utilis*	*S. cerevisiae*	*A. flavus*
**36a**	2	2	8	2	8
**Fluconazole**	1	4	8	16	256

Madabhushi et al. synthesized a new series of benzimidazole functionalized chiral thioureas and assessed for their antimicrobial activity against *S. aureus*, *B. subtilis*, *S. aureus* MLS16, *M. luteus*, *K. planticola*, *E. coli* and *P. aeruginosa*. Among them, compounds **37a** and **37b** displayed excellent antibacterial activity toward selected microorganisms (Table 36, Fig. 8) [45].

**Table 36:** Antibacterial activity of compounds (37a–37b)

Compounds	Microbial strains (MIC = µg/mL)						
	*S. aureus*	*B. subtilis*	*S. aureus MLS16*	*M. luteus*	*K. planticola*	*E. coli*	*P. aeruginosa*
**37a**	25.0	12.5	12.5	25.0	25.0	12.5	6.25
**37b**	25.0	12.5	12.5	6.25	12.5	12.5	6.25
**Neomycin**	12.5	12.5	12.5	12.5	12.5	12.5	12.5

**Fig. 9:**
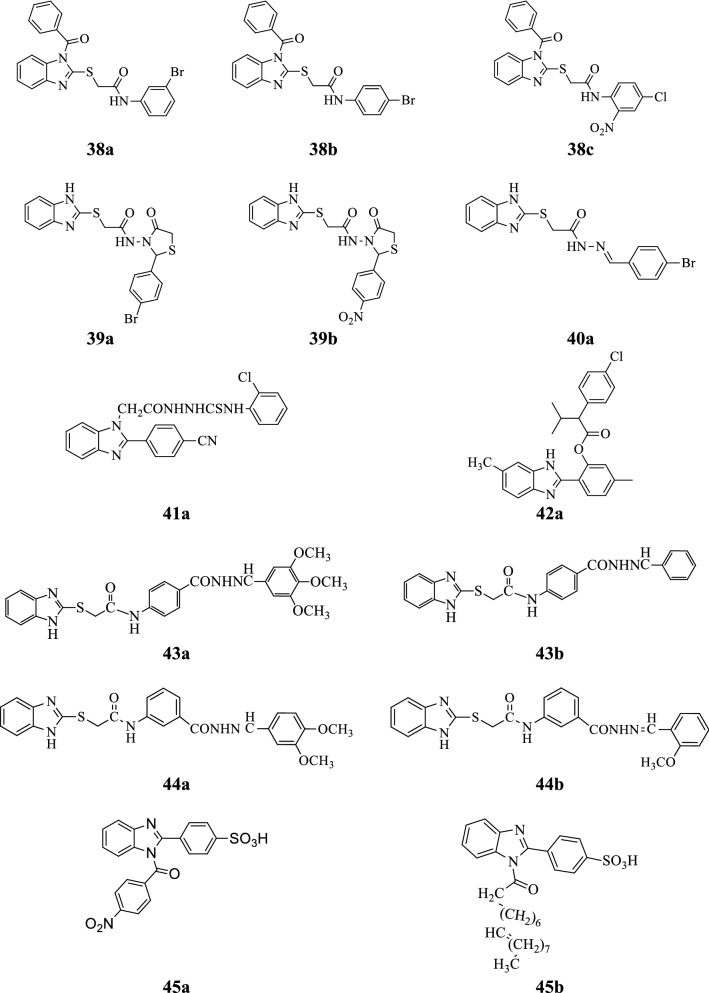
Molecular structures of compounds **(38a**–**38c**, **39a**–**33b**, **40a**, **41a**, **42a**, **43a**–**43b**, **44a**–**44b**, **45a–45b)**

Yadav et al. synthesized some 2-(1-benzoyl-1*H*-benzo[*d*]imidazol-2-ylthio)-*N*-substituted acetamide derivatives and evaluated for their antimicrobial activity (MIC and MBC/MFC) against tested strains by tube dilution method using cefadroxil and fluconazole as references. Among the synthesized compounds, **38a**, **38b** and **38c** emerged out as excellent antimicrobial agents (Tables 37, 38 and Fig. 9) [46].

**Table 37:** Antimicrobial activity of compounds (38a–38c)

Compounds	Microorganisms (MIC = µM/mL)						
	*S. aureus*	*B. cereus*	*B. subtilis*	*S. typhi*	*E. coli*	*C. albicans*	*A. niger*
**38a**	0.027	0.027	0.027	0.027	0.027	0.013	0.027
**38b**	0.027	0.027	0.027	0.027	0.027	0.013	0.027
**38c**	0.027	0.027	0.027	0.027	0.027	0.013	0.027
**Cefadroxil**	0.37	0.37	0.37	0.37	0.37	–	–
**Fluconazole**	–	–	–	–	–	0.47	0.47

**Table 38:** Antimicrobial activity (MBC/MFC) of compounds (38a–38c)

Compounds	Microorganisms (µg/mL)						
	*S. aureus*	*B. cereus*	*B. subtilis*	*S. typhi*	*E. coli*	*C. albicans*	*A. niger*
**38a**	50	> 50	> 50	> 50	> 50	50	> 50
**38b**	> 50	> 50	> 50	50	> 50	50	> 50
**38c**	50	> 50	50	50	50	> 50	> 50

Yadav et al. reported a class of novel benzimidazole derivatives and screened for its antimicrobial potency (MIC, MBC/MFC) against *S. aureus*, *B. subtilis*, *E. coli*, *C. albicans*, *A. niger* by tube dilution method using norfloxacin and fluconazole standard drugs. Compounds **39a** and **39b** showed prominent antimicrobial activity (Tables 39, 40 and Fig. 9) [47].

**Table 39:** Antimicrobial activity (MIC = µM/mL) of compounds (39a–39b)

Compounds	Microorganisms				
	*S. aureus*	*B. subtilis*	*E. coli*	*C. albicans*	*A. niger*
**39a**	0.027	0.027	0.013	0.027	0.027
**39b**	0.029	0.029	0.015	0.007	0.029
**Norfloxacin**	0.47	0.47	0.47	–	–
**Fluconazole**	–	–	–	0.50	0.50

**Table 40:** Antimicrobial activity (MBC/MFC) of compounds (39a–39b)

Compounds	Microorganisms (µg/mL)				
	*S. aureus*	*B. subtilis*	*E. coli*	*C. albicans*	*A. niger*
**39a**	> 0.108	> 0.108	0.013	0.054	0.054
**39b**	> 0.116	> 0.116	0.015	0.015	0.116

Yadav et al. designed a series of new benzimidazole derivatives and accessed for its antimicrobial potential against *S. aureus*, *B. subtilis*, *E. coli*, *C. albicans*, *A. niger* by tube dilution method. In this series, compound **40a** displayed the most potent antimicrobial activity (Table 41, Fig. 9) [48].

**Table 41:** Antimicrobial activity (MIC = µM/MBC/MFC = µg/mL) of compound 40a

Compound	Microorganisms				
	*S. aureus*	*B. subtilis*	*E. coli*	*C. albicans*	*A. niger*
**40a**	0.032/> 50	0.032/> 50	0.032/> 50	0.016/> 50	0.032/> 50
**Cefadroxil**	0.345	0.345	0.345	–	–
**Fluconazole**	–	–	–	0.40	0.82

Kerimov et al. developed new benzimidazole derivatives and evaluated for their antifungal activity against *C. albicans* and *C. krusei* by the agar diffusion method using fluconazole as standard. Among the synthesized compounds, compound **41a** (Table 42 and Fig. 9) found to be most active against tested fungal species [49].

**Table 42:** Antifungal activity of compound 41a

Compound	Fungal strains (ZI mm)	
	*C. albicans*	*C. krusei*
**41a**	15	15
**Fluconazole**	19	20

Si et al. synthesized a series of new benzimidazole scaffolds and evaluated for their antifungal activity against *Botrytis cinerea* and *Sclerotinia sclerotiorum* using thiabendazole and azoxystrobin as references. In this series, compound **42a** exhibited excellent antifungal activity (Table 43 and Fig. 9) [50].

**Table 43:** In vitro antifungal activity of compound 42a

Compound	Fungal strains [EC_50_ ± SE (mg/L)]	
	*B. cinerea*	*S. sclerotiorum*
**42a**	9.75 ± 0.23	18.27 ± 0.22
**Thiabendazole**	14.16 ± 0.20	39.43 ± 0.23
**Azoxystrobin**	39.22 ± 0.26	30.37 ± 0.28

Tahlan et al. reported a class of novel benzimidazole Schiff base derivatives and screened for its antimicrobial potency against tested microbial strains by tube dilution method. Among the synthesized compounds, **43a** and **43b** were found to be most potent antifungal agents against *A. niger* and *C. albicans* (Table 44 and Fig. 9) [51].

**Table 44:** Antimicrobial results of compounds (43a–43b)

Compounds	Microbial strains (MIC = µM/mL)						
	Bacterial strains					Fungal strains	
	*S. aureus*	*E. coli*	*B. subtilis*	*P. aeruginosa*	*S. enterica*	*C. albicans*	*A. niger*
**43a**	9.62	9.62	2.41	2.41	4.81	2.41	1.20
**43b**	5.82	2.91	5.82	5.82	5.82	1.46	2.91
**Cefadroxil**	1.72	1.72	1.72	1.72	1.72	–	–
**Fluconazole**	–	–	–	–	–	2.04	2.04

Tahlan et al. reported a series of new benzimidazole Schiff base derivatives and evaluated for its antimicrobial potency against selected microbial species. In this series, compounds **44a** and **44b** showed significant antimicrobial activity towards tested bacterial and fungal strains (Table 45 and Fig. 9) [52].

**Table 45:** Antimicrobial results of compounds (44a–44b)

Compounds	Microbial strains (MIC = µM/mL)						
	Bacterial strains					Fungal strains	
	*B. subtilis*	*P. aeruginosa*	*E. coli*	*S. typhi*	*K. pneumoniae*	*C. albicans*	*A. niger*
**44a**	1.28	1.28	1.28	2.55	5.11	5.11	2.55
**44b**	0.68	0.68	2.72	2.72	5.44	5.44	2.72
**Cefadroxil**	1.73	3.46	3.46	0.86	3.46	–	–
**Fluconazole**	–	–	–	–	–	4.08	4.08

Yadav et al. synthesized a series of novel benzimidazole derivatives and accessed for its antimicrobial activity against *S. aureus*, *B. subtilis*, *E. coli*, *C. albicans* and *A. niger* by serial dilution method using ciprofloxacin and fluconazole as standard drugs. From the synthesized derivatives, compounds **45a** and **45b** showed excellent antimicrobial activity against selected microorganisms (Tables 46, 47 and Fig. 9) [53].

**Table 46:** Antibacterial and antifungal activities of compounds (45a–45b)

Compounds	Microorganisms (pMIC = µM/mL)				
	*S. aureus*	*B. subtilis*	*E. coli*	*C. albicans*	*A. niger*
**45a**	2.43	2.43	2.43	2.13	1.53
**45b**	2.24	2.24	1.85	1.94	1.63
**Ciprofloxacin**	0.19	0.20	0.28	–	–
**Fluconazole**	–	–	–	0.20	0.22

**Table 47:** Antibacterial and antifungal activities of compounds (45a–45b)

Compounds	Microorganisms (MBC/MFC = µg/mL)				
	*S. aureus*	*B. subtilis*	*E. coli*	*C. albicans*	*A. niger*
**45a**	50	50	15.6	25	> 50
**45b**	12.5	50	3.12	50	> 50
**Ciprofloxacin**	0.019	0.019	0.019	–	–
**Fluconazole**	–	–	–	0.040	0.040

## Conclusions

Summarizingly, after review of literature reports we concluded that benzimidazole is most promising category of bioactive heterocyclic compound that exhibit a wide variety of biological activities i.e. antimicrobial, anti-inflammatory, antiparasitic, antimalarial, antiviral, antimycobacterial, antineoplastic, antihypertensive activity etc. The present review only focus on antimicrobial activity of reported benzimidazole derivatives may serve as valuable source of information for researchers who wish to synthesize new molecules of benzimidazole nucleus which have immense potential to be investigated for newer therapeutic possibilities. Condensed information of most active compounds with their antimicrobial activity and abbreviation of microbial species and other are shown in Tables 48 and 49, respectively.

**Table 48:**
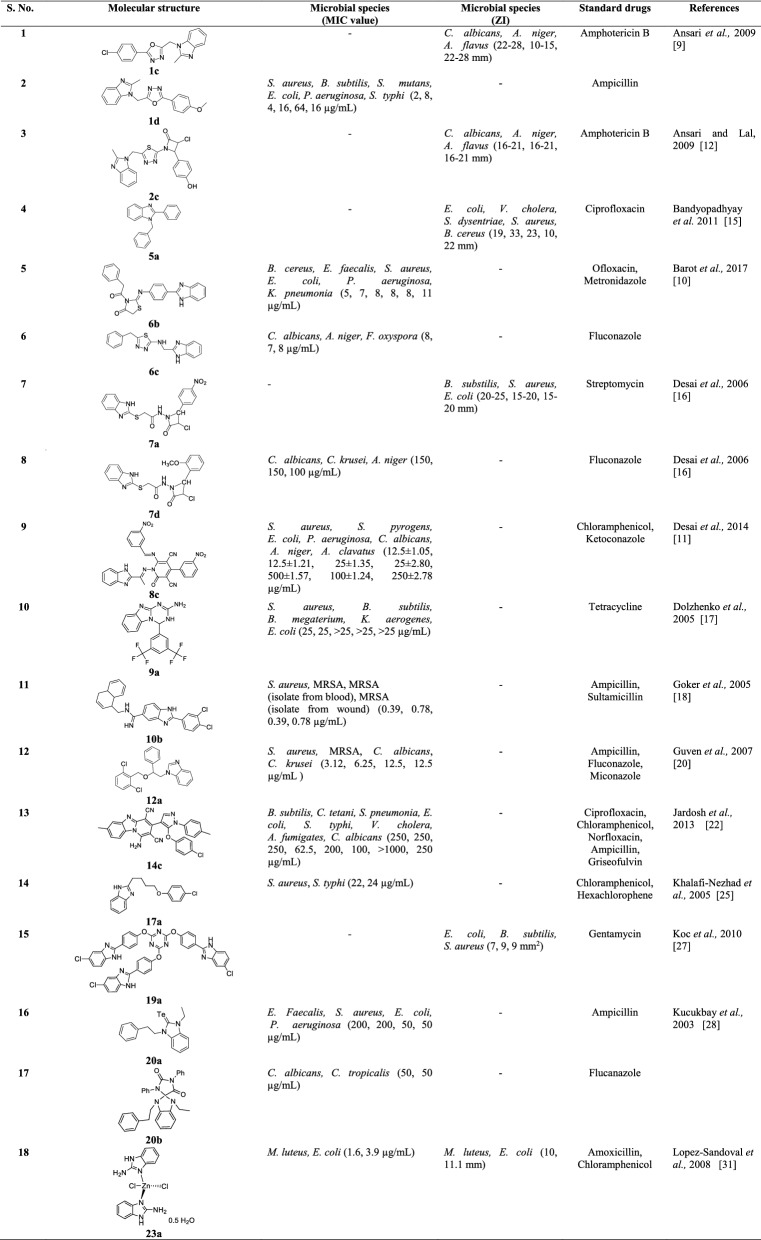

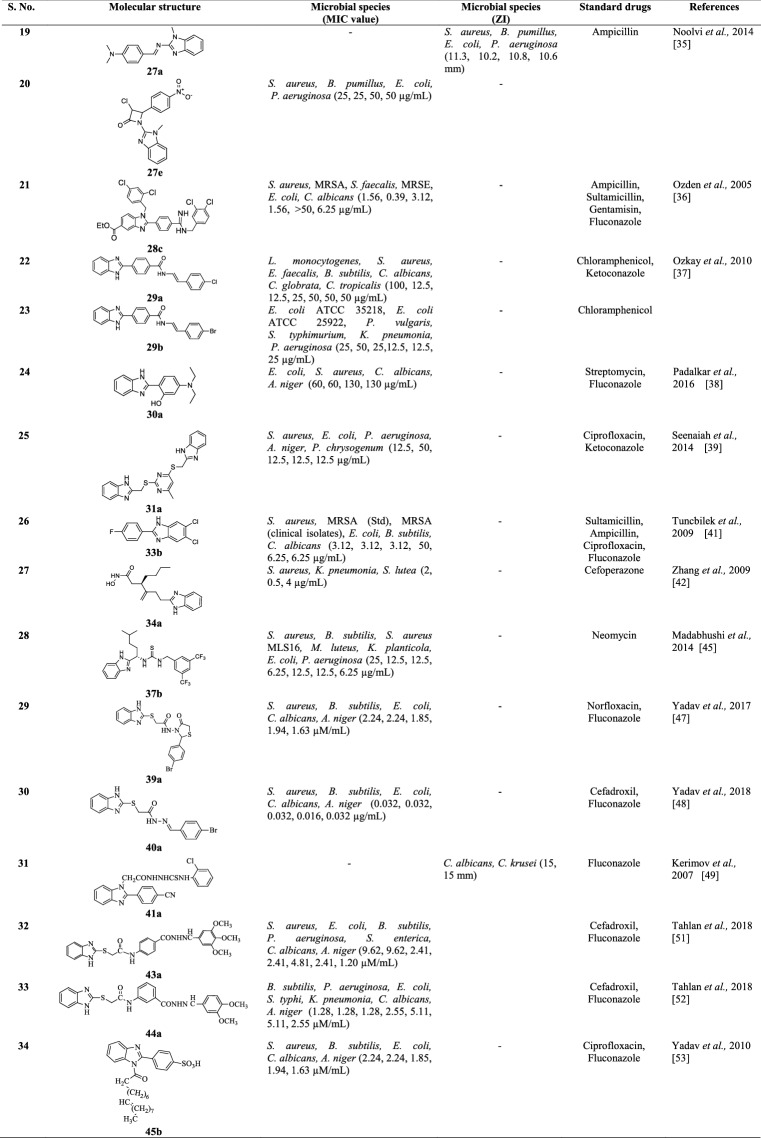
Condensed information of most active compounds with their antimicrobial activity

**Table 49:** Abbreviation of microbial species and other

*Absidia corymbifera*: *A. corymbifera*	Methicillin-resistant *Staphylococcus aureus*: MRSA
	Zone of inhibition: ZI
*Aspergillus clavatus*: *A. clavatus*	Methicillin-resistant *Staphylococcus epidermidis*: MRSE
*Aspergillus flavus*: *A. flavus*	Minimum inhibitory concentration: MIC
*Aspergillus fumigatus*: *A. fumigatus*	*Micrococcus luteus*: *M. luteus*
*Aspergillus niger*: *A. niger*	Multi-drug-resistant *Staphylococcus aureus*: MDRSA
*Bacillus cereus*: *B. cereus*	*Mycobacterium avium*: *M. avium*
*Bacillus megaterium*: *B. megaterium*	*Mycobacterium tuberculosis*: *M. tuberculosis*
*Bacillus proteus*: *B. proteus*	*Penicillium chrysogenum*: *P. chrysogenum*
*Bacillus pumilus*: *B. pumilus*	*Proteus vulgaris*: *P. vulgaris*
*Bacillus subtilis*: *B. subtilis*	*Pseudomonas aeruginosa*: *P. aeruginosa*
*Bacillus typhi*: *B. typhi*	*Rhizoctoni solani*: *R. solani*
*Botrytis cinerea*: *B. cinerea*	*Mycobacterium kansasii*: *M. kansasii*
*Candida albicans*: *C. albicans*	*Salmonella enterica*: *S. enterica*
*Candida glabrata*: *C. glabrata*	*Saccharomyces cerevisiae*: *S. cerevisiae*
*Candida krusei*: *C. krusei*	*Salmonella typhi*: *S. typhi*
*Candida mycoderma*: *C. mycoderma*	*Salmonella typhimurium*: *S. typhimurium*
*Candida tropicalis*: *C. tropicalis*	*Sarcina lutea*: *S. lutea*
*Candida utilis*: *C. utilis*	*Sclerotium rolfesii*: *S. rolfesii*
*Clostridium tetani*: *C. tetani*	*Shigella dysenteriae*: *S. dysentriae*
*Eberthella typhosa*: *E. typhosa*	*Staphylococcus aureus*: *S. aureus*
*Enterococcus faecalis*: *E. faecalis*	*Staphylococcus epidermidis*: *S. epidermidis*
*Escherichia coli*: *E. coli*	*Streptococcus faecalis*: *S. faecalis*
*Francisella tularensis*: *F. tularensis*	*Streptococcus mutans*: *S. mutans*
*Fusarium oxyspora*: *F. oxyspora*	*Streptococcus pneumoniae*: *S. pneumoniae*
*Fusarium solani*: *F. solani*	*Streptococcus pyogenes*: *S. pyogenes*
*Klebsiella aerogenes*: *K. aerogenes*	*Sclerotinia sclerotiorum*: *S. sclerotiorum*
*Klebsiella planticola*: *K. planticola*	Structure activity relationship: SAR
*Klebsiella pneumoniae*: *K. pneumoniae*	*Trichophyton mentagrophytes*: *T. mentagrophytes*
*Listeria monocytogenes*: *L. monocytogenes*	*Trichosporon beigelii*: *T. beigelii*
Minimum bactericidal concentration: MBC	Vancomycin-resistant *Enterococccus faecium*: VRE
Minimum fungicidal concentration: MFC	*Vibrio cholerae*: *V. cholera*
